# Association Study of *SLC6A4* (5-HTTLPR) Polymorphism and Its Promoter Methylation with Rehabilitation Outcome in Patients with Subacute Stroke

**DOI:** 10.3390/genes12040579

**Published:** 2021-04-16

**Authors:** Massimo Santoro, Mariacristina Siotto, Marco Germanotta, Alessia Mastrorosa, Dionysia Papadopoulou, Irene Aprile

**Affiliations:** IRCCS Fondazione Don Carlo Gnocchi ONLUS, 50143 Florence, Italy; masantoro@dongnocchi.it (M.S.); mgermanotta@dongnocchi.it (M.G.); amastrorosa@dongnocchi.it (A.M.); dpapadopoulou@dongnocchi.it (D.P.); iaprile@dongnocchi.it (I.A.)

**Keywords:** *SLC6A4*, 5-HTTLPR polymorphism, methylation, stroke, rehabilitation

## Abstract

Recently it has been suggested that *serotonin transporter* (*SLC6A4*) and its 5HTTLPR polymorphism could be involved in post stroke recovery. Here, we characterized the methylation profile of two different CpG islands within the *SLC6A4* promoter region in the whole blood of 50 patients with subacute stroke before and after a six-week rehabilitation treatment. These patients were genotyped for 5HTTLPR polymorphism identifying patients on the basis of short (S) and L (L) alleles: 17 patients LL, 22 patients LS and 11 patients SS. At baseline, all CpG sites for both CpG islands displayed a heterogeneous methylation percentage that were not influenced by the different genotypes. After rehabilitation, we found a significant variation in the methylation levels (increase/decrease) in the specific CpG sites of both CpG islands. The statistical analysis showed a significant relationship between the LL, LS and SS alleles and the outcome of the rehabilitation intervention (*χ*^2^ (2,50) = 6.395, *p* = 0.041). Specifically, we found a significant difference between patients with or without a favorable outcome in the LL (11.1% with a favorable outcome) and in the SS (54.4% with a favorable outcome) groups. Our data suggest that 5-HTTLPR polymorphisms and *SLC6A4* promoter methylation may be employed as a non-invasive biological marker of recovery in patients with stroke undergoing rehabilitation.

## 1. Introduction

Stroke is the primary cause of disability [1] with a heterogeneous clinical spectrum often linked also to incomplete recovery of motor function after a rehabilitation treatment [2]. For this reason, in recent years, the identification of biological markers of recovery after a stroke insult is emerging as an important research field, aimed to developing personalized rehabilitation programs. Current predictors used for post-stroke rehabilitation are the severity of the initial impairment [3,4], corticospinal tract integrity [5], and the location and measure of the lesion size [6,7]. Unfortunately, these factors explain only partially the variability observed in patients’ recovery [8].

Recently, several studies have shown how genetic and epigenetic factors can modulate the neurotransmission and neuroplasticity processes influencing the individual’s response to post-stroke rehabilitation treatments [9,10,11,12]. The serotonin transporter protein (5-HTT) has received particular attention because of its involvement in mood, depression, cognition, and brain development [13]. Indeed, 5-HTT influences the duration of serotonin action at the synapses, by causing a reuptake by the pre-synaptic neuron of serotonin itself from the synaptic cleft [11]. 5-HTT is encoded by *serotonin transporter* gene (*SLC6A4*, solute carrier family 6, member 4), located on chromosome 17q11.1–17q12; this gene consists of 13–14 exons spanning a genomic region approximately of 35 kilo bases (kb) [14]. *SLC6A4* expression is regulated by the biallelic polymorphism in its promoter region (5-HTTLPR) located upstream of the transcription start site (TTS) [14].

5-HTTLPR (rs4795541) polymorphism is a 44-base pair (bp) repeat insertion/deletion that generates long (L, with 16 repeats) and short (S, with 14 repeats) alleles, respectively [15,16,17]. Moreover, these two alleles show different transcriptional activity, with the S variant that reduces *SLC6A4* expression levels and serotonin uptake compared to the L variant [15,16,17]. Different studies showed that the S allele could be associated with some forms of dependence such as alcohol and heroin [18,19]. Studies on depression characterized the S variant as the sensitive allele due to its reduced ability to remove serotonin (5-HT) from the synaptic space, in comparison to the L allele [17,20].

However, the data are conflicting, since some studies found a significant association of the S allele with post-stroke depression [21,22], while another obtained the opposite results [23]. Moreover, *SLC6A4* expression is also significantly regulated by DNA methylation of cytosines in cytosine-guanine (CpG) dinucleotides. Several studies have focused on the possible association between peripheral *SLC6A4* methylation and brain processes. Indeed, Wang et al. found that the methylation of specific CpG sites in *SLC6A4* promoter region of T lymphocytes and monocytes from adults was associated with lower in vivo positron emission tomography (PET) measures of 5-HT synthesis in the orbitofrontal cortex, suggesting an association between peripheral DNA methylation and brain 5-HT synthesis [24]. Analyzing DNA from saliva and whole-blood samples in association with functional magnetic resonance imaging (fMRI) studies, Ismaylova et al. showed an association of peripheral *SLC6A4* methylation with the prefrontal cortical volume and parietal-frontal regional functional connectivity [25].

Moreover, hypermethylation of the *SLC6A4* promoter region has been correlated with low levels of *SLC6A4* expression in psychiatric disorders such as alcoholism, and depression [26]. In post-stroke patients, high methylation levels of the *SLC6A4* promoter have been associated with both post-stroke depression (PSD) and worsening of depressive symptoms suggesting that DNA methylation might modulate the response to stress with implications for physical function and quality of life [27]. Early antidepressant treatment of PSD appears to enhance both physical and cognitive recovery from stroke and might increase survival up to 10 years following stroke [28].

Moreover, neuropsychological deficits have significant impacts on functional recovery, quality of life and sociality and could negatively influence rehabilitation strategy [29].

However, it must be considered that these associations with the different levels of methylation may be affected by the presence of variants in DNA sequence, such as 5HTTLPR polymorphism. For instance, in patients with PSD the association of *SLC6A4* methylation status with depression was significantly higher in participants carrying the 5-HTTLPR SS genotype [27]. In individuals with unresolved trauma, the SS genotype was associated with lower methylation levels of *SLC6A4* promoter regions compared to the LL genotype [30]. This evidence supports a role for methylation in the modulation of the response to external or internal stress with implication on cognitive and physical function. So, it is possible to hypothesize that *SLC6A4* methylation status and 5-HTTLPR polymorphism could be implicated in post-stroke recovery after rehabilitation treatment.

In order to characterize this issue, we analyzed the methylation levels of two CpG islands located in the *SLC6A4* promoter region on DNA from whole blood of patients with subacute stroke. Finally, we correlated the data obtained with the 5-HTTLPR polymorphism and methylation with rehabilitation outcome before and after treatment.

## 2. Materials and Methods

### 2.1. Sample

In this study, we enrolled 50 consecutive patients admitted to our rehabilitation department after a subacute stroke. Inclusion criteria were: (i) first ischemic or hemorrhagic stroke, confirmed by Magnetic Resonance Imaging (MRI) or Computed Tomography (CT); (ii) age between 55 and 85 years; (iii) patients able to perform a rehabilitation treatment, for at least 45 min/day, for 5 days/week; (iv) time since the acute event within 6 months; (v) cognitive and language abilities sufficient to understand the experiments and follow instructions. Exclusion criteria were: (i) a previous stroke; (ii) behavioral and cognitive disorders and/or reduced compliance interfering with active therapy.

All patients gave written informed consent after a detailed explanation of the study’s aims and rehabilitation protocols. The institutional Ethics and Experimental Research Committee approved the study protocol on 13 March 2019 (FDG_6_13/3/19) that was registered on Clinicaltrial.gov (ClinicalTrials.gov Identifier: NCT04223180) (accessed on 26 February 2021).

Demographic and clinical data (kind and side of stroke, latency form onset, spatial neglect, language impairment and pharmacologic therapy) were reported in Table 1.

Depression was evaluated in all patients using the Back Depression Scale (BDS). Scores from 0 through 9 indicate no or minimal depression; scores from 10 through 18 indicate mild to moderate depression; scores from 19 through 29 indicate moderate to severe depression; and scores from 30 through 63 indicate severe depression [31].

### 2.2. Rehabilitation Treatment and Outcome

Patients underwent a rehabilitation program including both conventional and robotic physiotherapy. The conventional physiotherapy, including passive mobilizations, stretching, sensory stimulation, task practice, and functional training, was performed six times/week, each session lasting 45 min, and focused on lower limbs, sitting and standing training, balance, and walking. Robotic treatment of the upper limb was performed 5 times a week, each session lasting 45 min using a set of robotic devices: Motore (Humanware, Pisa, Italy), and Amadeo, Diego and Pablo (Tyromotion, Graz, Austria). Patients were evaluated at the admission (T0) and re-evaluated after 6 weeks of rehabilitation treatment (T1) by means of the modified Barthel Index (BI), an ordinal scale used to measure performance in activities of daily living (ADL), ranging from 0 to 100, with lower scores indicating increased disability [32].

### 2.3. DNA Extraction

Genomic DNA was extracted from venous blood of all patients with stroke at T0 and T1 using Quick-DNA Midiprep Plus Kit (Zymo Research, Irvine, CA, USA) and quantified by the Qubit 2.0 Fluorometer (ThermoFisher Scientific, Waltham, MA, USA) according to the manufacturer’s instructions.

### 2.4. SLC6A4 (5-HTTLPR) Genotyping

5-HTTLPR genotyping was performed using polymerase chain reaction (PCR) polymerase chain reaction (PCR) based on fragment length polymorphism. The PCR fragments were amplified from 20 ng of genomic DNA (reaction volume of 20 μL), using MyTaq DNA polymerase kit (Bioline, Memphis, TN, USA) and 10mM of each primers previous described [27] which flank the genomic region containing the 5-HTTLPR polymorphism. The PCR conditions were as follows: 2 min at 95 °C, initial denaturation; 15 s at 95 °C, 45 s at 60 °C and 1 min at 72 °C for 35 cycles; 7 min at 72 °C, final extension. The PCR products were resolved by electrophoresis on 2% agarose gels stained with Gel Red (Biotium, Fremont, CA, USA) in order to identified PCR fragments of different sizes: (i) short (S) 486 base pair (bp) with 14 repeats; (ii) long (L) 529 bp with 16 repeats; (iii) extra-long (XL) 612/654 bp with 20/22 repeats.

### 2.5. DNA Bisulphite Conversion and SLC6A4 Methylation Analysis

**Figure 1:**
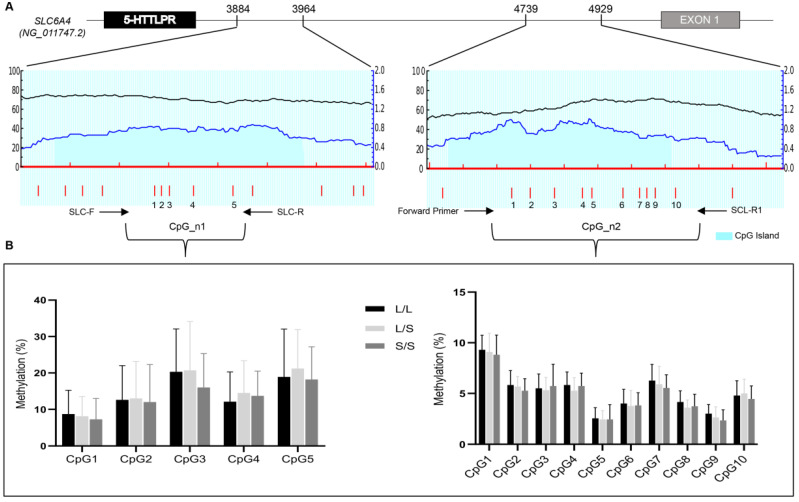
Methylation analysis of *SLC6A4*. (**A**) Upper panel: schematic representation of *SLC6A4* promoter region. Gray box as exon 1, black box as 5-HTTLPR polymorphism location, and intronic sequences as thick black line. Lower panel: CpG island (blue region) identified by MethPrimer program [33]. The first CpG island indicated as CpG_n1 is located at the end of the 5-HTTLPR polymorphism (accession number: NG_011747.2, position 3884–3964). The second CpG island indicated as CpG_n2 is the promoter region previously described [27] (accession number: NG_011747.2, position 4739–4929). Vertical red bars indicate relative positions of CpG sites that are numbered from 1 to 5 for CpG_n1 island and from 1 to 10 for CpG_n2 island. The PCR primers used in this study are indicated as arrows. (**B**) Histogram of the average methylation percentage in patients with stroke carrying the LL (Long Long), LS (Long Short), and SS (Short Short) alleles for each CpG island.

The methylation study was performed on two CpG islands identified through the online platform MethPrimer [33] (Figure 1A).

The first CpG island (indicated as CpG_n1) was located at the end of the 5-HTTLPR polymorphism (accession number: NG_011747.2, position 3884–3964) containing 5 CpG sites (Figure 1A). The second CpG island (indicated as CpG_n2) was the promoter region previous described [27] (accession number: NG_011747.2, position 4739–4929) including 10 CpG sites (Figure 1A). Then, 2 μg of genomic DNA was bisulphite converted using EZ DNA Methylation- Gold Kit (Zymo Research, Irvine, CA, USA) according to the manufacturer’s instructions. After bisulphite treatment, the genomic DNA was quantified by the Qubit 2.0 Fluorometer (ThermoFischer Scientific, Waltham, MA USA) ac Bioline cording to the manufacturer’s instructions. An 80 bp fragment of CpG_n1 region was amplified by PCR from bisulfite-treated DNA using HS MyTaq DNA polymerase (Bioline, Memphis, TN, USA), a forward primer SLC-F (5′-GGCGTTTAGGTGGTATTAGAAT-3′) and a reverse primer SLC-R (5′-biotinylated-CTAAACTAAACAACCACGAACAA 3′). For the CpG_n2 region, a 189 bp fragment was amplified using a forward primer previous described [27] and SLC-R1 (5′-biotinylated-CCTAACTTTCCTACTCTTTAACTTTAC-3′). For both amplicons, the PCR conditions were as follows: 95 °C for 15 min, followed by 50 cycles of 95 °C for 15 s, 60 °C for 30 s, and 70 °C for 30 s with final extension of 10 min at 72 °C.

Pyrosequencing was performed on a PyroMark Q24 pyrosequencer (Qiagen, Hilden, Germany), with the following sequencing primers: SLC-F for CpG_n1 region while SLC-S1 (5′-TAGGAAGAAAGAGAGAG-3′) and SC-S2 (5′-GAGTAGATTTTTGTGTGT-3′) for CpG_n2 region. The percentage of methylation on each CpG region was quantified using the PyroMark Q24 software v2.0.7 (Qiagen, Hilden, Germany). All experiments were performed in triplicate and data were expressed as the mean ± standard error (SE).

### 2.6. Statistical Analysis

The demographic and clinical characteristics of the enrolled sample are described as means and standard deviations, or percentage, as appropriate. The mean values of the methylation percentages in patients with different genotypes (LL, LS and SS) were compared by using non-parametric Kruskal–Wallis tests, followed by post-hoc comparisons with Bonferroni correction, when necessary. For pairwise comparisons, the adjusted *p*-values (i.e., the uncorrected *p*-value multiplied by 3, with three being the number of comparisons made) are reported. The analyses were conducted for all the investigated sites, separately.

To investigate the changes in the methylation percentages that occurred during the investigated timeframe, we compared, for each methylation site, the values obtained at T0 with those at T1 by using Wilcoxon Rank Sum tests. For the subsequent analyses, only methylation sites showing a statistically significant change were considered.

To investigate the relationship between 5-HTTLPR polymorphism, *SLC6A4* promoter methylation and the outcome of the rehabilitation intervention, we dichotomized the outcome of the rehabilitation treatment, i.e., the modified Barthel Index (BI) after the rehabilitation intervention, into favorable and unfavorable. The BI cut-off scores were defined as BI ≥ 75 for a favorable outcome and as BI < 75 for an unfavorable outcome [34].

Then, we evaluated: (a) the relationship between the three genotypes (LL, LS, and SS) and the rehabilitation outcome (favorable and unfavorable) through a chi-squared test, followed by a post-hoc test, and (b) the relationship between the methylation percentage before and after the rehabilitation intervention, as well as their changes from baseline and the rehabilitation outcome (favorable and unfavorable) through Mann–Whitney U tests.

For each statistical analysis, a *p*-value lower than 0.05 was deemed significant. Statistical analysis was performed using SPSS version 25 (IBM Corp., Armonk, NY, USA), while figures were made using GraphPad Prism version 8 (GraphPad Software, San Diego, CA, USA).

## 3. Results

### 3.1. Samples

In this study, we analyzed 50 patients undergoing a rehabilitation intervention. All patients were clinically evaluated at the enrollment (T0) and after a 6-week rehabilitation treatment (T1); blood samples of all 50 patients were taken at T0, while blood samples of 36 patients (72%) were taken at T1.

Table 1 reports demographic, clinical characteristics and anti-depressive treatment with selective serotonin reuptake Inhibitors (SSRI) of patients with stroke enrolled in this study. Thirty-four patients (68.0%) showed a moderate to severe depression (19 ≤ BDI ≤ 29), while in 16 patients (32.0%) a severe depression was detected (BDI ≥ 30).

**Table 1:** Demographic and clinical characteristics of the sample (*n* = 50) at enrollment (T0).

Variable	Mean (SD), or Count (%)
Age	68.7 (14.3)
Sex	27 men (54.0%) 23 women (46.0%)
Time since stroke (days)	89.7 (31.1)
Type of stroke	37 ischemic (74.0%) 13 hemorrhagic (26.0%)
Hemiparesis side	20 right (40.0%) 30 left (60.0%)
Spatial Neglect	10 (20.0%)
Language impairment	9 (18.0%)
SSRI	26 (52.0%)
Modified Barthel Index	38.5 (17.7)

### 3.2. 5-HTTLPR Genotype and Methylation Analysis

Genotyping analysis for 5-HTTLPR polymorphism was performed on DNA from peripheral blood of 50 patients with stroke using PCR amplification [27].

We identified 17 (34%) patients carrying homozygous LL genotype, 22 (43.1%) patients with heterozygous LS genotype, and 11 (21.6%) patients with homozygous SS genotype. Methylation analysis was carried out on two CpG islands indicated as CpG_n1 and CpG_n2 that contained 5 CpG sites and 10 CpG sites respectively (Figure 1A).

At baseline, we found in the CpG_n1 island an average methylation level of 14.5% in patients with LL genotype, 15.3% in patients with LS genotype, and 13.7% in patients with SS genotype. However, all CpG sites displayed heterogeneous methylation levels (8–22%) that were not influenced by the different genotypes, being the *p* values always higher than 0.05 (Figure 1B left panel). Conversely, considering the CpG_n2 island we detected an average methylation level of 5.2% in patients with the LL genotype, 4.9% in patients with the LS genotype, and 4.8% in patients with the SS genotype. Even in this case, the analyzed CpG sites showed different methylation levels (2–9%) that were not influenced by the different genotypes (*p* values > 0.05) (Figure 1B right panel).

**Figure 2:**
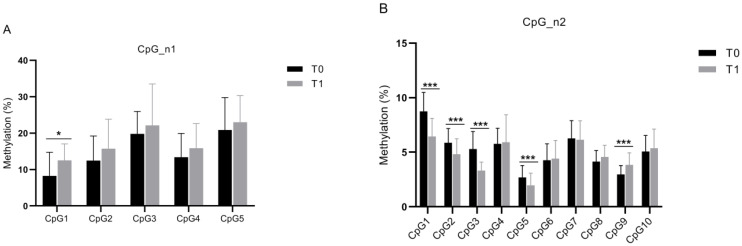
Histogram of *SLC6A4* average methylation percentage. (**A**) Methylation degree of five CpG sites within CpG_n1 island analyzed in 36 patients with stroke at baseline (T0) and after the 30-session rehabilitation treatment (T1). (**B**) Methylation degree of ten CpG sites within CpG_n2 island analyzed in 36 patients with stroke at baseline (T0) and after the 30-session rehabilitation treatment (T1). The asterisks indicate a statistically significant differences: *** *p* < 0.001 and * *p* < 0.005, according to the paired *t*-tests.

Finally, we compared in 36 patients the methylation levels at T0 vs. T1 of the CpG_n1 and CpG_n2 islands. In the CpG_n1 island, statistical analysis showed a significant increase (~4%) in the CpG1 site only (*p* < 0.04) (Figure 2A). With respect to the CpG_n2 island, we found a decrease (~2–3%) in four out of ten sites (CpG1, CpG2, CpG3, *p* < 0.001; and CpG5, *p* = 0.007), while a statistically significant increase (~2%) was noted in the CpG9 site (*p* < 0.001) (Figure 2B).

### 3.3. Correlation of 5-HTTLPR Genotype/Methylation Analysis and Rehabilitation Outcome

**Figure 3:**
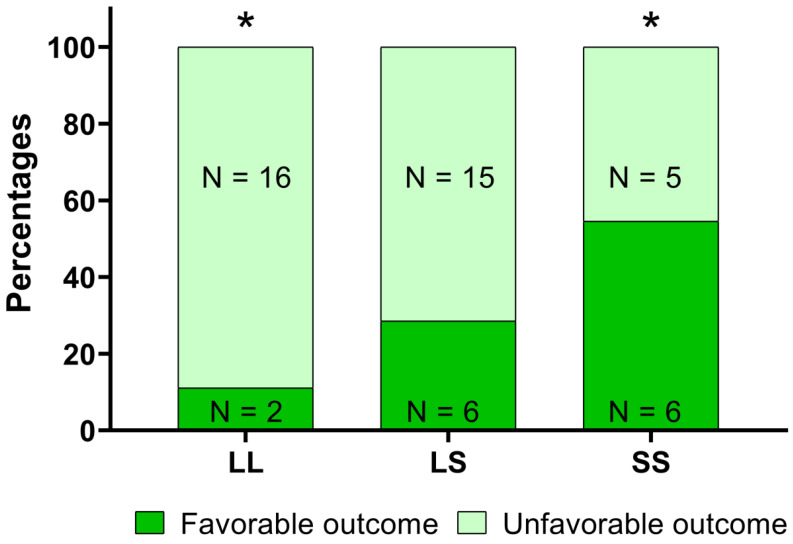
Analysis of 5-HTTLPR polymorphism and the rehabilitation outcome. In the figure, the percentages (together with the absolute numbers, N) of patients with or without a favorable outcome after a 6 week rehabilitation intervention, i.e., a score in the modified Barthel Index equal to or higher than 75, for patients with different 5-HTTLPR polymorphisms, are reported. According to the ***χ***^2^ test, there was a significant relationship between the polymorphisms and the rehabilitation outcome. The asterisks are related to the results of the post-hoc analysis and indicate a *p* value lower than 0.05.

Our results showed a significant relationship between the LL, LS, and SS alleles and the outcome of the rehabilitation intervention [χ^2^ (2,50) = 6.395, *p* = 0.041, Figure 3].

The post-hoc analysis, with Bonferroni’s correction, highlighted a statistically significant difference between patients with or without a favorable outcome in the LL (11.1% of patients with a favorable outcome) and the SS groups (54.4% of patients with a favorable outcome). Considering the methylation data, we found in CpG_n1 island that the CpG1 site at T1 showed a methylation level significantly higher in patients with a favorable outcome; similarly, we detected in patients with a favorable outcome a higher increase of methylation level of CpG4 and CpG5 sites between T0 and T1 (Table 2).

On the contrary, in the CpG_n2 island we found significantly lower methylation levels of the CpG1, CpG2 and CpG5 sites in patients with a favorable outcome, when compared to those with an unfavorable outcome (Table 3).

## 4. Discussion

Patients with subacute stroke present a heterogeneous motor disability implying that the recovery of motor function after the rehabilitation program can be variable, and often incomplete. This variability could be influenced by genetic and epigenetic components that play an important biological role in cortical plasticity and neuronal processes modulating the response to post-stroke rehabilitation [10,12,35].

*SLC6A4* is the gene coding for the serotonin transporter involved in the serotonin reuptake from the synapse, and is a key regulator of serotonergic neurotransmission.

The *SLC6A4* gene expression is regulated by both the biallelic 5-HTTLPR polymorphism and CpG methylation at the promoter region [15,36]. It is not surprising that the *SLC6A4* gene has been studied extensively in recent years.

In our study, we found a significant relationship between the LL, LS and SS genotypes and rehabilitation outcome [*χ*^2^ (2,50) = 6.395, *p* = 0.041, Figure 3].

We found that 54.4% of patients carrying the SS allele had a favorable outcome, while only 11.1% of patients carrying LL alleles obtained the same results. These different responses to rehabilitation treatment could be explained considering that the 5-HTTLPR S allele is involved in the regulation of *SLC6A4* expression and amygdala activation [37,38] that could affect the patient’s emotional reactivity to a specific treatment.

Moreover, it is important to consider that disability at the baseline was not significantly different between patients carrying different alleles.

On the other hand, *SLC6A4* expression levels were regulated by epigenetic chromatin remodeling such as the DNA methylation of CpG sites in its promoter region [36].

Our study focused on analyzing methylation levels of the CpG sites in the genomic region immediately downstream of the 5-HTTLPR polymorphism, indicated as CpG_n1 island, and in the promoter region, indicated as CpG_n2, previously studied by Kim and collaborators [27] (Figure 1A). All CpG sites displayed heterogeneous methylation levels with 8–22% for CpG_n1 island and 2–9% for CpG_n2 island, that were not influenced by the different genotypes (*p* values > 0.05) (Figure 1B).

The methylation results obtained for CpG sites in CpG_n2 region appear to be at odds with those from Kim et al., who found a significantly higher methylation percentage of SS alleles compared to LL or LS alleles [27]. This incongruence could be related to a difference in the enrollment period of the patients. In fact, while Kim et al. analyzed the methylation levels of samples recruited respectively after two weeks and after one year from the stroke event, in our study, we enrolled patients within six months since stroke. A difference in the latency from the stroke insult could probably play a functional role in modulating the methylation levels of the *SLC6A4* promoter.

Moreover, we found a significant variation in the methylation levels (increase/decrease) of both CpG_n1 and CpG_n2 islands of the *SLC6A4* promoter region after rehabilitation treatment (Figure 2B) while there were no significant differences at baseline. This result should be confirmed and possibly reinforced with a larger number of subjects undergoing rehabilitation treatment.

Finally, comparing the BI with the different genotype, we found a statistically significant difference between patients with or without a favorable outcome in the LL (11.1% of patients with a favorable outcome) and the SS groups (54.4% of patients with a favorable outcome). Moreover, if we consider methylation data of both CpG_n1 and CpG_n2 islands, we found a significantly variation of methylation levels (increase/decrease) between patients with or without a favorable outcome at T1 respect to baseline (Table 2 and Table 3).

Our study, characterizing the peripheral methylation of the *SLC6A4* promoter region in stroke patients after a rehabilitation program, extends the relevance of serotonergic neurotransmission to rehabilitation outcome and to the response modulation to external or internal stimuli, with repercussions on physical function.

This turns out to be particularly interesting if we consider that the peripheral DNA methylation of specific CpG sites in the *SLC6A4* promoter region is associated with lower in vivo 5-HT synthesis in the orbitofrontal cortex measured by PET, suggesting peripheral *SLC6A4* methylation as a potential biomarker of central serotonin function [24].

On the basis of this evidence, it is possible to hypothesize that an increase or decrease of the *SLC6A4* methylation levels, with a consequent alteration of the gene expression, could imply an alteration of 5-HT recycling and homeostasis leading to different serotonin concentration in the synaptic cleft. First of all, the relationship between serotonin alteration and PSD is an important topic to consider, being that PSD appears to negatively influence both physical and cognitive recovery from stroke [28,29].

Moreover, the 5-HT system is involved in the neurogenesis [39,40] and in the interaction with the hypothalamic–pituitary–adrenal (HPA) axis [41,42], whose endocrine changes represent the first alterations caused by the ischemic stroke [43,44,45].

Although the functional role of 5-HTT methylation remains highly speculative, our findings support a role for *SLC6A4* promoter methylation in stroke recovery; an implication of this results to verify in further studies could be the use of 5-HTT methylation as prognostic biomarker for long-term rehabilitation, and in conjunction with 5-HTTLPR polymorphism for favorable and unfavorable outcomes.

It is important to note that we have also characterized a new genomic region (CpG_n1) located immediately downstream of the 5-HTTLPR and near others functional polymorphisms such as rs25531/rs25532, both located in the L and S alleles [46] which can modulate the allele frequency variation and serotonin transporter functionality [47,48]. So, the functional role and the interactive effect of 5-HTTLPR/rs25531/rs25532 polymorphisms with *SLC6A4* promoter methylation could be characterized in future studies in subjects affected by depression, or by alcohol or drug dependence and under antidepressants.

This pilot study has important limitations: the small sample size and the fact that we obtained blood samples at T1 only for 72% of subjects. For this reason, further studies are necessary in a cohort with a greater number of subjects allowing a better characterization of the 5-HTTLPR polymorphism and its methylation in relation to the type of stroke (ischemic or hemorrhagic), or the stroke severity. Moreover, our study considers a mean latency since stroke of 90 days and, therefore, further study in earlier phases of stroke recovery should be designed.

However is important to highlight that the present study has presented, for the first time, a correlation analysis of the 5-HTTLPR polymorphism and its methylation with rehabilitation outcomes.

## 5. Conclusions

If the preliminary results of this study are confirmed, *SLC6A4* could become a reliable prognostic genetic/epigenetic factor used to better identify optimal treatments for a patient, or to supplement rehabilitation therapy with a customized rehabilitation protocol, along with the *BDNF* rs6265 polymorphism recently conceived from our group [12].

In this way, *SLC6A4* could be used as non-invasive biomarker in post-stroke patient management, with personalized rehabilitation protocols reducing costs and recovery times.

## Figures and Tables

**Table 2:** Methylation percentage comparison of the CpG_n1 island sites (CpG1, CpG2, CpG3, CpG4, CpG5) at baseline (T0), after rehabilitation treatment (T1) and their changes from baseline (Δ) in patients with or without a favorable outcome (BI ≥ 75 at discharge). *p* values refer to the Mann–Whitney U test.

CpG_n1 Sites	Patients with Unfavorable Outcome Mean (SD)	Patients with Favorable Outcome Mean (SD)	*p*
CpG1 (T0)	8.5 (6.4)	7.6 (4.1)	0.974
CpG2 (T0)	13.7 (10.7)	10.5 (6.7)	0.509
CpG3 (T0)	21.6 (13.0)	15.4 (7.5)	0.124
CpG4 (T0)	14.5 (8.9)	11.1 (5.6)	0.346
CpG5 (T0)	21.5 (12.5)	16.1 (4.9)	0.243
CpG1 (T1)	9.9 (4.2)	12.9 (5.1)	**0.049**
CpG2 (T1)	15.7 (6.8)	15.9 (7.4)	0.751
CpG3 (T1)	21.5 (11.0)	24.3 (13.2)	0.537
CpG4 (T1)	15.3 (6.7)	18.0 (7.0)	0.320
CpG5 (T1)	22.1 (7.1)	26.3 (7.6)	0.168
Δ CpG1	1.4 (8.6)	5.4 (5.7)	0.339
Δ CpG2	2.6 (11.3)	5.6 (11.3)	0.641
Δ CpG3	0.6 (17.8)	8.3 (12.2)	0.236
Δ CpG4	0.7 (9.6)	8.6 (6.1)	**0.024**
Δ CpG5	−0.5 (13.0)	11.4 (7.5)	**0.009**

Bold values indicate a statistically significant difference between the two groups (*p* < 0.05).

**Table 3:** Methylation percentage comparison of the CpG_n2 island sites (CpG1, CpG2, CpG3, CpG4, CpG5)at baseline (T0), after rehabilitation treatment (T1) and their changes from baseline (Δ) in patients with or without a favorable outcome (BI ≥ 75 at discharge). *p* values refer to the Mann–Whitney U test.

CpG_n2 Sites	Patients with Unfavorable Outcome Mean (SD)	Patients with Favorable Outcome Mean (SD)	*p*
CpG1 (T0)	9.2 (1.7)	9.0 (1.8)	0.598
CpG2 (T0)	5.8 (1.3)	5.3 (1.0)	0.101
CpG3 (T0)	5.5 (1.5)	5.4 (1.6)	0.851
CpG5 (T0)	2.4 (1.0)	2.4 (1.3)	0.441
CpG9 (T0)	2.8 (.9)	2.3 (1.1)	0.097
CpG1 (T1)	6.9 (1.6)	5.0 (1.2)	**0.004**
CpG2 (T1)	4.9 (1.0)	3.6 (0.7)	**0.004**
CpG3 (T1)	3.4 (0.8)	2.9 (0.6)	0.135
CpG5 (T1)	1.9 (1.3)	0.8 (0.7)	**0.027**
CpG9 (T1)	3.8 (1.1)	4.1 (1.2)	0.320
Δ CpG1	−2.1 (2.0)	−3.0 (1.8)	0.193
Δ CpG2	−1.1 (1.4)	−1.9 (1.1)	0.070
Δ CpG3	−2.0 (1.9)	−1.9 (1.5)	0.867
Δ CpG5	−0.9 (1.5)	−2.1 (1.2)	0.107
Δ CpG9	0.7 (1.1)	1.5 (1.2)	0.070

Bold values indicate a statistically significant difference between the two groups (*p* < 0.05).

## Data Availability

The data that support the findings of this study are available from the corresponding author upon reasonable request.
